# The minor allele of rs17427875 in long non-coding RNA-HOXA11-AS influences the prognosis of subarachnoid hemorrhage (SAH) via modulating miR-15a and STAT3 expression

**DOI:** 10.18632/aging.204126

**Published:** 2022-06-14

**Authors:** Yong Zhou, Zhiming Xu, Shengli Li

**Affiliations:** 1 Neurosurgical Department, Qingdao Municipal Hospital, Qingdao 266011, China

**Keywords:** subarachnoid hemorrhage, lncRNA HOXA11-AS, SNP, miR-15a, STAT3

## Abstract

Background: HOAX11-AS was reported to promote the progression of liver cancer via the signaling pathway of miR-15a-3p/STAT3. In this study, we investigated the effect of rs17427875 on the prognosis of subarachnoid hemorrhage (SAH) and its underlying molecular mechanisms.

Methods: 158 SAH patients were recruited and grouped according to their genotypes rs17427875. Peripheral blood and cerebrospinal fluid (CSF) samples were collected for subsequent analysis. Quantitative real-time PCR, luciferase assays, Western blot and ELISA were performed to analyze the correlations between the expression of lncRNA-HOXA11-AS, miR-15a, *TNF-α* and *NF-κB*.

Results: The survival rate was remarkably higher in SAH patients carrying the AA genotype of rs17427875 when compared with those carrying the AT genotype. The expression of miR-15a was significantly repressed in the peripheral blood and CSF of SAH patients carrying the AT allele when compared with that in patients carrying the AA allele. MiR-15a showed a remarkable efficacy in inhibiting the luciferase activity of wild type lncRNA-HOXA11-AS and STAT3 in THP-1 cells. P-HOXA11-AS-T showed a stronger ability to suppress the expression of miR-15a and activate the expression of *STAT3*, *TNF-α* and *NF-κB* in THP-1 cells when compared with P-HOXA11-AS-A.

Conclusions: The findings demonstrated that the presence of the minor allele of rs17427875 in lncRNA-HOXA11-AS could increase the expression level of lncRNA-HOXA11-AS, thus elevating the expression level of STAT3 via down-regulating miR-15a, and increased STAT3 expression could aggravate inflammation to cause poor prognosis of SAH. Therefore, the rs17427875 polymorphism can be used as a potential biomarker for the prognosis of SAH.

## INTRODUCTION

Typically appearing as a result of the rupture of a vascular malformation or an arterial aneurysm, SAH tends to have poor prognosis. The occurrence of cerebral ischemia at several days after the onset of SAH has actually been linked to vasoconstriction as well as exacerbated damages to the neurologic system [1]. Therefore, the study of the molecular mechanism underlying the pathology of cerebral ischemia may produce targets for the treatment of SAH.

Long non-coding RNAs (LncRNAs) are a type of RNA transcripts that are over 200 nt in length and have no protein transcription ability [2, 3]. LncRNAs have been shown to be associated with a number of cellular processes, such as the differentiation of normal cells as well as the progression of cancer cells [4–6]. HOMEOBOX A11 (HOXA11) antisense RNA (HOXA11-AS) is a newly found lncRNA shown to manage the development of glioma cells by competing with other gene transcripts for miR-214-3p binding [7, 8]. HOXA11-AS was shown to participate in the carcinogenic processes of a variety of human cancers such as non-small cells lung cancer, colon cancer, as well as gastric cancer [9, 10]. In addition, the overexpression of HOXA11-AS was found to promote the proliferation and growth of liver tumor cells [11]. As a component in the miR-15a/16 cluster, miR-15a is encoded by a gene whose expression in leukemia was deleted [12, 13]. MiR-15a was shown to act as a tumor promoter or tumor suppressor in several kinds of tumors, such as prostate cancer, lymphocytic lymphoma, as well as pituitary adenomas [14]. In cervical cancer cases, the up-regulated expression of miR-15a-3p enhances the sensitivity of cancer cells to radiotherapy by controlling the protein expression of D5217. MiR-15a-3p expression is down-regulated in gastric cancer to prevent the metastasis of gastric cancer through blocking Twist1 expression [15]. A past study showed that HOXA11-AS can work as a competing endogenous RNA (ceRNA) of miR-15a-3p. It was further shown that the expression of miR-15a-3p was down-regulated in liver cancer by HOXA11-AS [16].

STAT3 was initially as a member in the acute phase response factor (APRF) complex activated by interleukin-6 by doing a crucial job in activating innate immunity in the liver. This discovery of STAT3 aided the identification of a series of STAT proteins that can respond to interferon (IFN) [17–19]. In another study, STAT3 was verified to act as a miR-15a-3p target. Furthermore, the results of the study also validated the presence of a negative regulatory relationship between miR-15a-3p and STAT3 as well as the presence of a positive regulatory relationship between miR-15a-3p and HOXA11-AS [16]. It was noted that in the rats of an experimental SAH model, the levels of IL-6 as well as JAK2 expression were notably increased in cerebral arteries [20]. Since JAK2 and IL-6 are essential to the activation of STAT3, the levels of phosphorylated STAT3 was also notably increased at 6 h after the onset of SAH. Nevertheless, another recent research revealed that the treatment of SAH mice using inhibitors of phosphorylated STAT3 had little impact on the neurologic results of SAH [21]. It was previously revealed that the expression of minor and common rs17427875 alleles of HOXA11-AS at the ectopic level suppressed the development of tumor phenotypes. Particularly, the minor allele of rs17427875 displayed a higher level of tumor suppressing activity [22].

The rs17427875 polymorphisms in HOXA11-AS has been reported to be correlated with the susceptibility to various diseases [22, 23]. For example, the presence of T allele of rs17427875 improve the function of HOXA11-AS as a tumor suppressor in epithelial ovarian cancer [20], and the presence of T allele of rs17427875 was also associated with reduced risk of lung adenocarcinoma as a protective factor for lung cancer [23]. Moreover, HOAX11-AS was reported to promote the progression of liver cancer via the signaling pathway of miR-15a-3p/STAT3 [16]. Therefore, in this study, we hypothesized that the minor allele of rs17427875 located in HOAX11-AS could exhibit beneficial effect upon the risk of SAH, thus functioning as a biomarker for the prognosis of SAH.

## RESULTS

### Patient characteristics

In this study, we recruited 158 patients of SAH (Subarachnoid hemorrhage) and collected their peripheral blood and CSF (cerebrospinal fluid) samples. Genotyping was performed to determine the genotypes of rs17427875 in all patients. Then, the patients were divided into two groups according to their genotypes of rs17427875: AA (*N* = 138) and AT (*N* = 20). The information of the patients including their sex, smoking history, alcohol drinking history, and history of arterial hypertension and aneurysm was summarized in Table 1. Student’s *t* test indicated no obvious difference was observed between the two groups in terms of these patient characteristics.

**Table 1:** Demographic and clinical characteristics of participants of this study.

**Characteristics**	**AA (*N* = 138)**	**AT (*N* = 20)**	***P* value**
Sex, Female/Male	83/55	14/6	0.835
Smoking, Ever/Never	78/60	12/8	0.954
Alcohol, Ever/Never	71/67	12/8	0.285
Arterial hypertension, Yes/No	53/85	8/12	0.556
Aneurysm location, ACA/MCA/ICA/VBA	55/36/22/25	10/6/2/2	0.255

### The survival rate was remarkably elevated in SAH patients carrying the AA genotype of rs17427875

**Figure 1:**
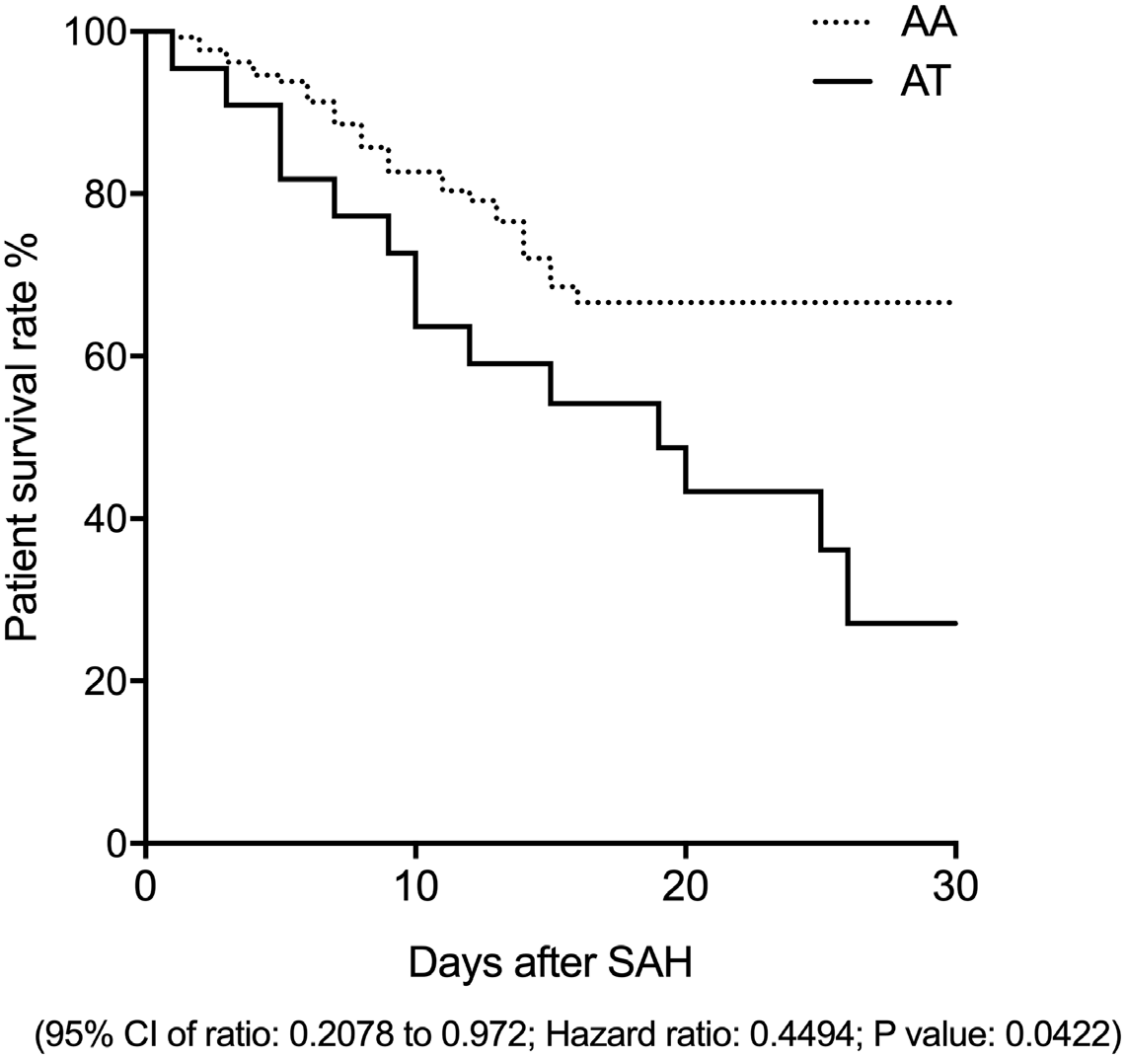
The survival rate of SAH patients carrying the AA genotype of rs17427875 was evidently higher than the patients carrying the AT genotype (OR = 2.1; 95 CI: 1.05-4.32; *P* value < 0.05).

A survival analysis was carried out for all patients at 30 days after the diagnosis of SAH. As shown in Figure 1, the survival rate was significantly increased in patients carrying the AA allele of rs17427875.

### Differential expression of lncRNA-HOXA11-AS, miR-15a, *TNF-α* and *NF-κB* in the peripheral blood of SAH patients carrying different genotypes of rs17427875

**Figure 2:**
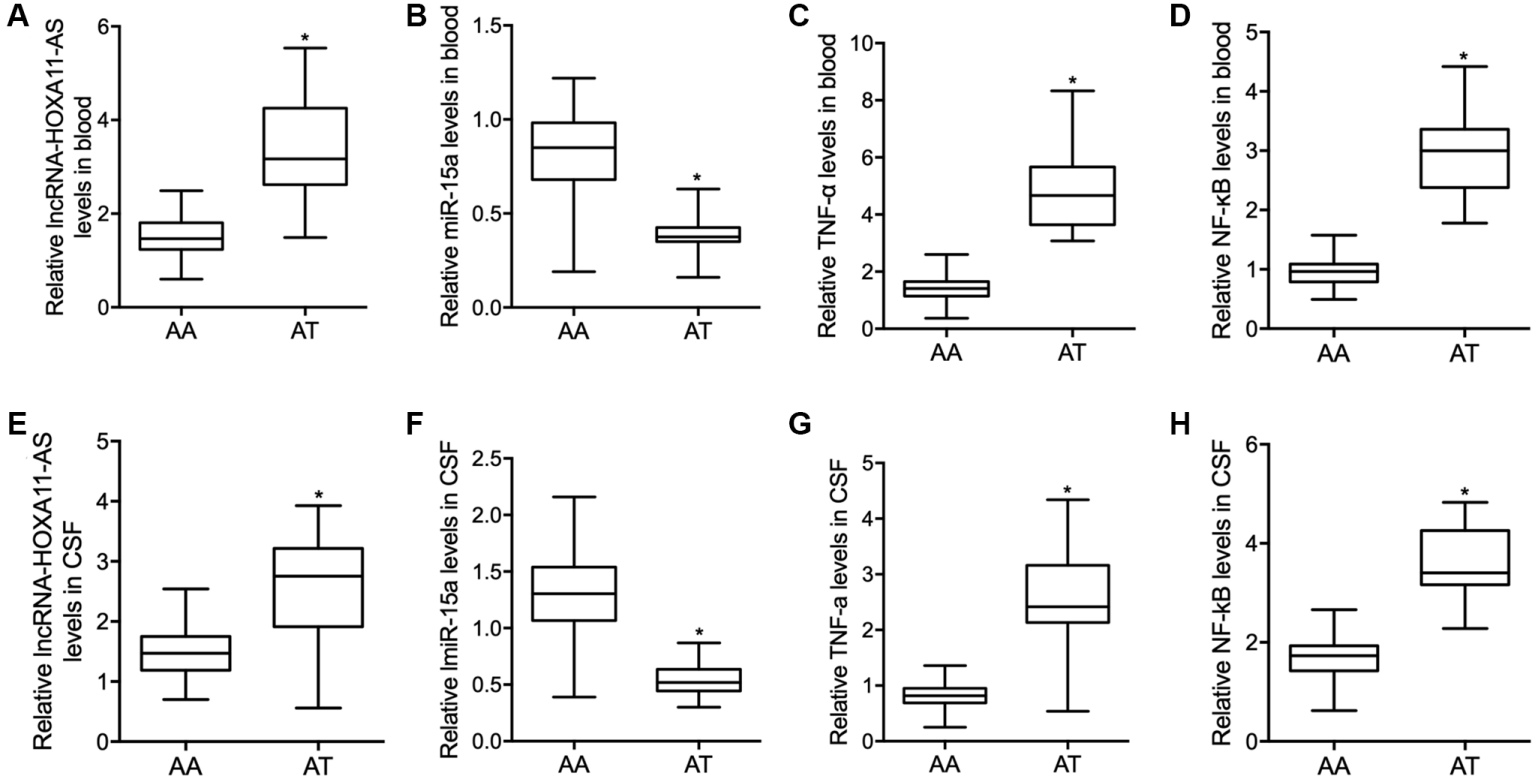
Compared with SAH patients carrying the AT genotype of rs17427875, in the peripheral blood samples, the relative expressions of lncRNA-HOXA11-AS (**A**) was significantly increased while the relative expression level of miR-15a (**B**) was notably repressed, and both the TNF-α (**C**) and *NF-κB* (**D**) level were significantly increased in SAH patients carrying the AT genotype of rs17427875. Moreover, similar results were observed of lncRNA-HOXA11-AS (**E**), miR-15a (**F**), TNF-α (**G**) and *NF-κB* (**H**) in the CSF samples collected from SAH patients carrying the AT genotype of rs17427875 (^*^P value < 0.05 vs. AA group).

Quantitative real time PCR was performed to examine the expression of lncRNA-HOXA11-AS, miR-15a, *TNF-α* and *NF-κB* in the peripheral blood collected from the SAH patients carrying different genotypes of rs17427875. The expression of lncRNA-HOXA11-AS was notably increased in the peripheral blood of SAH patients carrying the AT allele of rs17427875 when compared with that in patients carrying the AA allele (Figure 2A). On the contrary, the expression of miR-15a was remarkably suppressed in the peripheral blood of SAH patients carrying the AT genotype of rs17427875 (Figure 2B). The expression of *TNF-α* (Figure 2C) and *NF-κB* (Figure 2D) was apparently increased in the peripheral blood of SAH patients carrying the AT genotype of rs17427875.

### Differential expression of lncRNA-HOXA11-AS, miR-15a, *TNF-α* and *NF-κB* in the CSF of SAH patients carrying different genotypes of rs17427875

The expression of lncRNA-HOXA11-AS, miR-15a, *TNF-α* and *NF-κB* was further analyzed in the CSF collected from SAH patients carrying different genotypes of rs17427875. The expression of lncRNA-HOXA11-AS was notably increased in the CSF of SAH patients carrying the AT allele of rs17427875 when compared with the patients carrying the AA allele (Figure 2E). On the contrary, the expression of miR-15a was remarkably suppressed in the CSF of SAH patients carrying the AT genotype in comparison to patients carrying the AA genotype of rs17427875 (Figure 2F). The expression of *TNF-α* (Figure 2G) and *NF-κB* (Figure 2H) was apparently increased in the CSF of SAH patients carrying the AT genotype.

### The luciferase activity of lncRNA-HOXA11-AS and *STAT3* was suppressed by miR-15a in THP-1 cells

**Figure 3:**
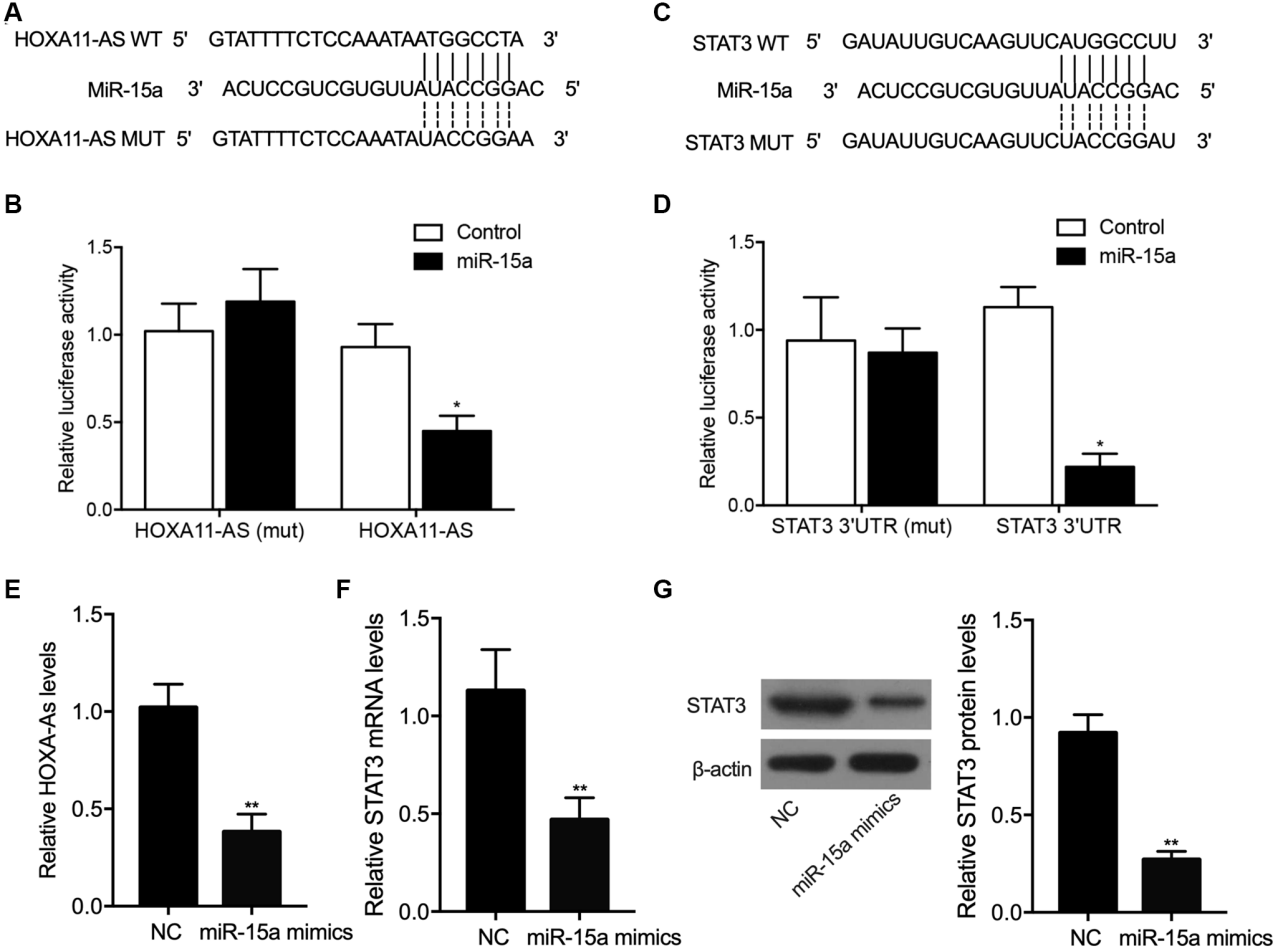
Sequence analysis showed that miR-15a could potentially target lncRNA-HOXA11-AS (**A**), and the transfection of miR-15a suppressed the luciferase activity of wild-type lncRNA-HOXA11-AS (**B**) in THP-1 cells. Moreover, sequence analysis showed that miR-15a could potentially target the 3’UTR STAT3 (**C**), and the transfection of miR-15a suppressed the luciferase activity of wild-type STAT3 (**D**) in THP-1 cells. Meanwhile, the transfection of miR-15a mimics significantly repressed the relative expression of HOXA11-AS (**E**), STAT3 mRNA (**F**) and protein (**G**) (^*^P value < 0.05 vs. control; ^**^P value < 0.05 vs. NC group).

Our binding site screening showed that miR-15a could potentially target lncRNA-HOXA11-AS (Figure 3A) and *STAT3* (Figure 3C). Luciferase vectors containing wild type and mutant lncRNA-HOXA11-AS and *STAT3* were established and transfected into THP-1 cells with miR-15a mimics. The luciferase activity of wild type lncRNA-HOXA11-AS (Figure 3B) and *STAT3* (Figure 3D) was remarkably suppressed by miR-15a, whereas no inhibition was observed for mutant lncRNA-HOXA11-AS and *STAT3*. Moreover, upon the transfection of miR-15a, the expressions of lncRNA-HOXA11-AS (Figure 3E), as well as the expressions of STAT3 mRNA (Figure 3F) and protein (Figure 3G) were significantly reduced. These results demonstrated that miR-15a could repress the expression of lncRNA-HOXA11-AS and *STAT3*.

### LncRNA-HOXA11-AS-T dramatically suppressed the expression of miR-15a and increased the expression of *STAT3* in THP-1 cells

**Figure 4:**
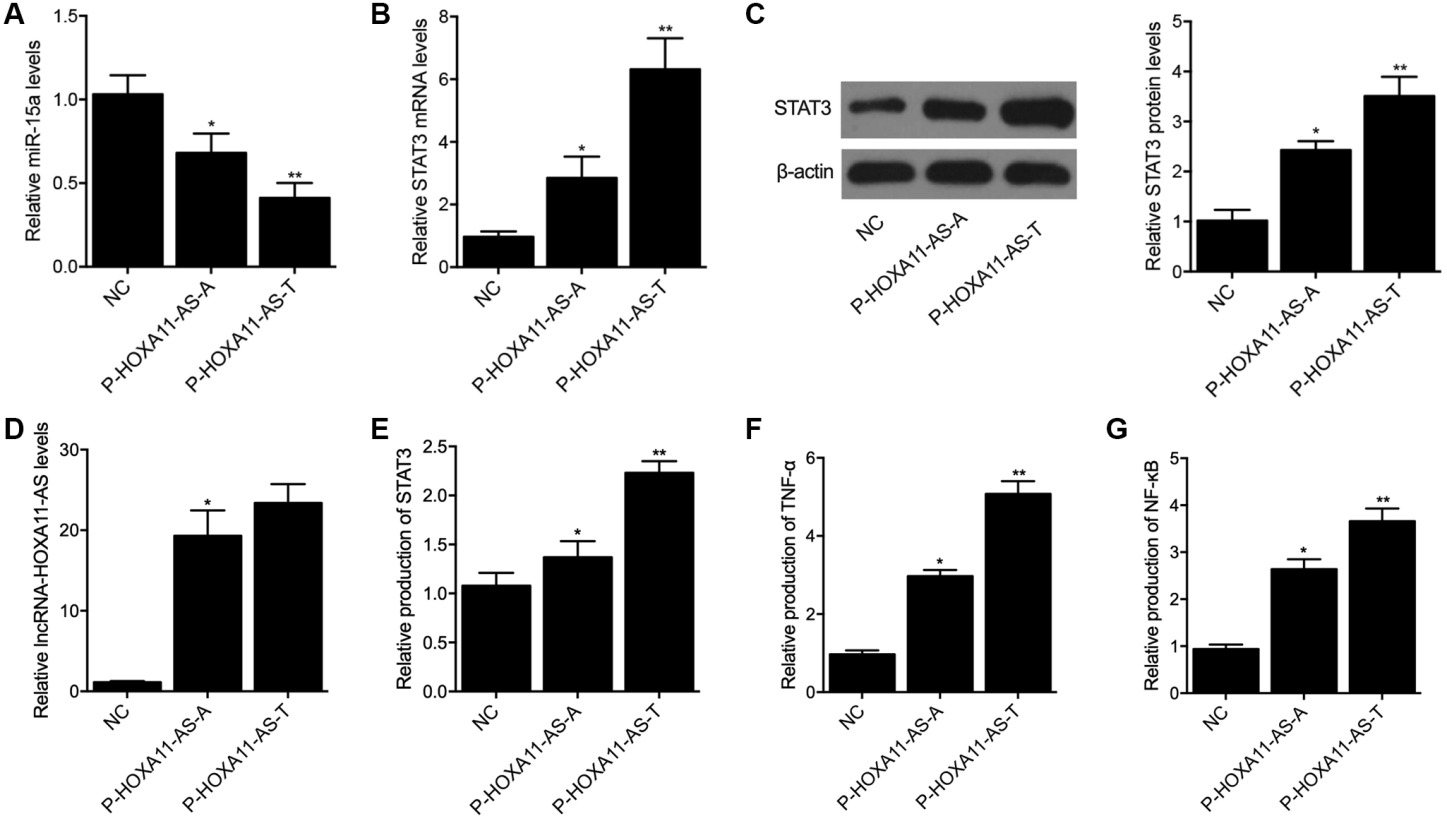
Compared with the A allele located in HOXA11-AS, the T allele located in HOXA11-AS showed a stronger capability to suppress the expression of miR-15a (**A**) and enhance the expression of STAT3 mRNA (**B**) and protein (**C**), as well as HOXA11-AS (**D**) in THP-1 cells. Meanwhile, ELISA analysis indicated that P-HOXA11-AS-T exhibited a stronger capability to increase the expression of STAT3 (**E**), TNF-α (**F**) and NF-κB (**G**) in THP-1 cells (^*^P value < 0.05 vs. NC group; ^**^P value < 0.05 vs. *P*-HOXA11-AS-A group).

In order to further explore the regulatory relationship among lncRNA-HOXA11-AS, miR-15a and *STAT3*, P-HOXA11-AS-A and P-HOXA11-AS-T were transfected into THP-1 cells, respectively. The expression of miR-15a and *STAT3* was examined in THP-1 cells transfected with P-HOXA11-AS-A and P-HOXA11-AS-T. The expression of miR-15a in THP-1 cells transfected with P-HOXA11-AS-A was remarkably suppressed when compared with the THP-1 cells transfected with the NC control. Moreover, the suppression of miR-15a expression was even more apparent in THP-1 cells transfected with P-HOXA11-AS-T (Figure 4A). On the contrary, the expression of STAT3 mRNA (Figure 4B) and protein (Figure 4C), as well as the expression of lncRNA-HOXA11-AS (Figure 4D), was progressively increased in THP-1 cells transfected with P-HOXA11-AS-A and P-HOXA11-AS-T.

### LncRNA-HOXA11-AS-T significantly increased the expression of STAT3, *TNF-α* and *NF-κB* in THP-1 cells

ELISA was carried out to evaluate the expression of STAT3, TNF-α and NF-κB in THP-1 cells transfected with P-HOXA11-AS-A and P-HOXA11-AS-T. As shown in Figure 4, the expression of STAT3 (Figure 4E), TNF-α (Figure 4F) and NF-κB (Figure 4G) in THP-1 cells was notably increased by P-HOXA11-AS-A, whereas P-HOXA11-AS-T showed a stronger capability to enhance the expression of STAT3, TNF-α and NF-κB in THP-1 cells.

## DISCUSSION

HOXA11-AS is located on the anti-sense strand of HOXA11-AS gene and its expression levels are different in tissues of rectal cancer, gastric cancer, as well as cervical cancer than that in normal tissues [8, 10, 24]. HOXA11-AS may also be involved in the progression of cervical cancer through regulating the gene expression of HOXAA [2, 25]. Rs17427875, an exotic mutation in the HOXA11-AS gene, has been recognized to reduce the risk of serous epithelial ovarian cancer (EOC) [22]. Edward et al. showed that the rs17427875-T genotype of HOXA11-AS was related to a reduced sensitivity to EOC [22]. It was also discovered that both the rs11564004-T and rs17427875-T polymorphisms of HOXA11-AS were linked to an increased risk of lung cancer. Moreover, in a past research, HOXA11-AS was shown to facilitate the expression of genes related to cell migration, proliferation as well as inflammation, thus stimulating inflammation in patients with diabetic arteriosclerosis [8, 26]. In this study, we recruited 158 SAH patients and divided them into two groups according to their genotypes of rs17427875. The survival analysis showed that the survival rate of patients carrying the AA allele was significantly higher than the patients carrying the AT genotype.

In addition, the peripheral blood and CSF samples were collected from SAH patients carrying different genotypes of rs17427875. The expression of lncRNA-HOXA11-AS, miR-15a, *TNF-α* and *NF-κB* was analyzed using qPCR. It has been reported that NF-κB is the main transcription factor which induced inflammation-related genes in IA lesions [27]. Also, the high expression of serum TNF-α was found to be associated with poor prognosis of SAH [28]. In this study, the expression of lncRNA-HOXA11-AS, *TNF-α* and *NF-κB* was remarkably increased in the peripheral blood and CSF of SAH patient carrying the AT genotype of rs17427875, while the expression of miR-15a was notably repressed in the peripheral blood and CSF of SAH patient carrying the AT genotype of rs17427875.

The overexpression of HOXA11-AS was shown to be associated with a poor prognosis of liver cancer patients by promoting the metastasis as well as growth of liver cancer. Furthermore, HOXA11-AS acts as a ceRNA targeting miR-15a-3p [16]. At the same time, miR-15a-3p can suppress the expression of its target STAT3. In this study, a luciferase assay was performed to explore the regulatory relationship between lncRNA-HOXA11-AS and miR-15a, as well as between miR-15a and STAT3. The luciferase activity of wild type lncRNA-HOXA11-AS and STAT3 was obviously suppressed by miR-15a in THP-1 cells. In addition, we transfected P-HOXA11-AS-A and P-HOXA11-AS-T into THP-1 cells and then detected the expression of miR-15a, STAT3, *TNF-α* and *NF-κB*. P-HOXA11-AS-T showed a stronger capability to suppress the expression of miR-15a and increase the expression of STAT3, *TNF-α* and *NF-κB* in THP-1 cells. The family members of STAT include 7 proteins that share certain conserved domains [29]. In particular, STAT3 is enriched in cells undergoing proliferation as well as differentiation [30]. While the activation of STAT3 is usually transient under normal conditions, STAT3 activation tends to become more persistent in a number of hematopoietic malignancies, such as multiple myeloma, melanoma, ovarian cancer, as well as prostate cancer [31]. In addition, the activation of ERK1/2 by STAT3 usually involves the two sites of Tyr705 and Ser727 [32, 33]. A previous study demonstrated quick STAT3 activation in the endothelial cells as well as smooth muscle cells of SAH rats. Especially, JAK1 as well as STAT3 phosphorylation at Tyr705 increased the concentration of IL-6 in CSF, whereas dramatically increased STAT3 phosphorylation at Ser727 was observed 1–2 days later [33]. Moreover, it remains controversial as whether the activation of microglial STAT3 leads to inflammation, although several recent researches actually revealed that STAT3 expression can be suppress the expression of IL-6 as well as anti-inflammatory IL-10, so as to exert both anti-inflammatory and pro-inflammatory effects [15, 34–36]. In the 1990’s, it was reported that the severity of cerebral vasospasm was closely related to the changes in the levels of inflammatory cytokines in the CSF, so as to induce secondary post-SAH brain injury [37, 38]. In the early 2000s, it was already reported that SAH causes systemic inflammations as well as modulation of the peripheral immunity [38, 39].

Despite the findings of this study demonstrated the role of minor allele of rs17427875 in lncRNA-HOXA11-AS in the pathogenesis of SAH, our conclusions are limited by the following two aspects: one is the lack of appropriate animal study to verify the findings of our study, and the other one is the small sample size we recruited for our human study. In our future investigations, a higher sample size with various originalities for the human study is expected. And the findings will accordingly be verified by an appropriate animal model.

**Figure 5:**
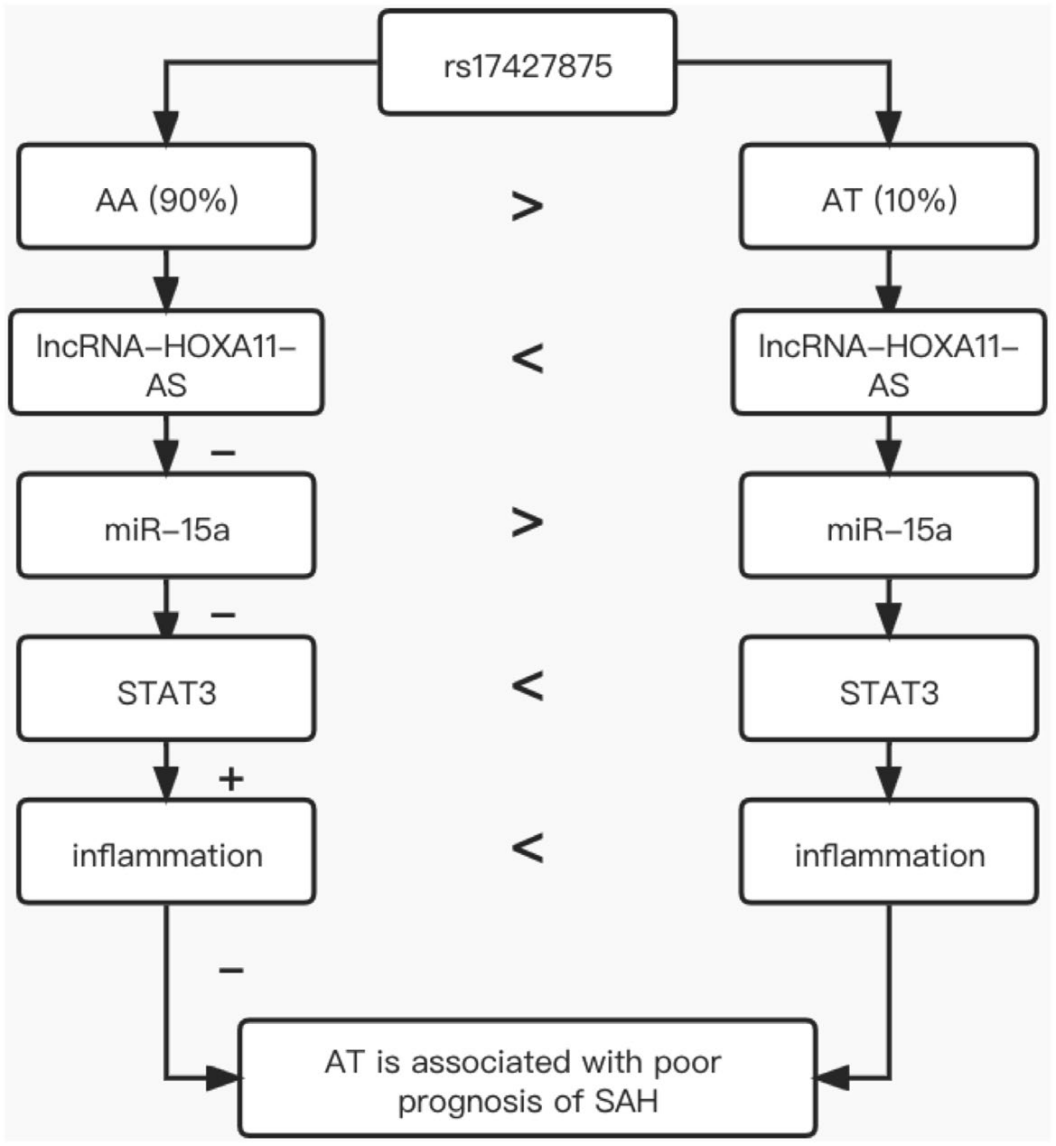
The underlying signaling mechanism discovered in our investigation.

In conclusion, this study demonstrated that the minor allele of rs17427875 in lncRNA-HOXA11-AS could increase the expression of lncRNA-HOXA11-AS, thus elevating the expression of STAT3 mRNA and protein via down-regulating miR-15a expression, and increased STAT3 expression could aggravate inflammation to cause poor prognosis of SAH (Figure 5). Therefore, the rs17427875 polymorphism can be used as a potential biomarker for the prognosis of SAH.

## MATERIALS AND METHODS

### Human subjects and sample collection

In this study, we recruited 158 SAH (Subarachnoid hemorrhage) patients and collected their peripheral blood and CSF (cerebrospinal fluid) samples. During the patient recruitment process, only those with aneurysmal SAH were enrolled in this study, and all those with SAH caused by trauma, glioma, other types of stroke, or other medical conditions of CNS were excluded from this study. In the next step, genotyping was performed (the details of genotyping were shown below) to determine the genotype of rs17427875 in each patients. Then, the patients were divided into two groups according to their genotypes of rs17427875, i.e., a AA group (*N* = 138, SAH patients carrying the AA genotype of rs17427875) and a AT group (*N* = 20, SAH patients carrying the AT genotype of rs17427875). The information of all patients including their sex, smoking history, alcohol drinking history, and history of arterial hypertension and aneurysm was collected and compared, and the Student’s *t* test was used to compare the differences between the two groups in terms of the above patient characteristics. In this study, the qualified patients were enrolled without considering their clinical status of SAH. This research was reviewed and approved by the Clinical Ethical Committee of our institute, while signed informed consent forms were collected from all subjects before this study was started. In this study, the measurements of TCD data were carried out according to a previous published methods [24]. In brief, all participants underwent CT examinations to verify their diagnosis of SAH based on the Fisher scale [25]. The pathogenesis of aneurysm was studied by using digital subtraction angiography or CT angiography. During the examinations, the aneurysm was fixed by using an intravascular coiling. Subsequently, CT scans were carried out after an oral administration of 60 mg/day of nimodipine for 6 days to prevent cerebral vasospasm.

### Genotyping by direct sequencing

In this study, for the collection operation of peripheral blood and CSF samples, all pipette tips as well as centrifuge tubes used in the procedure were free of RNase and DEPC-treated. In addition, all buffers used in the collection of peripheral blood and CSF samples were free of RNase and constituted using ddH_2_O treated with DEPC. To determine the genotypes of rs17427875 SNP in lncRNA-HOXA11-AS of each patient, total RNA was first extracted from each peripheral blood or CSF sample by using a Trizol reagent (Invitrogen, Carlsbad, CA, USA) according to instructions of the manufacturer. In the next step, poly (A) RNA was separated from around 200 μg of extracted total RNA of each sample by making use of an Oligotex mRNA mini assay set (Qiagen, Germantown, MD, USA) following the instructions of the kit. Then, a T4 RNA ligase (Takara, Tokyo, Japan) was used in accordance with the recommended assay protocol provided on the manual of the reagent to ligate the 3’ region of a 5’ RNA adapter to the free 5’ monophosphate site in the poly (A) RNA, and the product of ligation was purified by making use of the Oligotex mRNA mini assay kit in accordance with the recommended manual of the assay kit. In the next step, the purified RNA was subject to reverse transcription by using a Superscript II reverse transcriptase (Invitrogen, Carlsbad, CA, USA) in conjunction with Oligo (dT) primers in accordance with the recommended assay protocol provided by the manufacturer. Then, the obtained cDNA was amplified using PCR with Ex Taq DNA Polymerase (Takara, Tokyo, Japan) in accordance with the recommended assay protocol provided by the manufacturer. Finally, the PCR product was subjected to direct sequencing on an Illumina HiSeq2000 direct sequencer (Illumina, San Diego, CA, USA) in accordance with the recommended protocol provided by the instrument manufacturer.

### RNA isolation and real-time PCR

In this study, quantitative real time PCR was performed to examine the expression of lncRNA-HOXA11-AS, miR-15a, *TNF-α* and *NF-κB* in the peripheral blood and CSF samples collected from the SAH patients carrying different genotypes of rs17427875. In brief, the total RNA content in each cell and tissue sample was extracted by making use of the Oligotex mRNA mini assay set according to the kit’s instruction. Then, an MMLV High Performance Reverse Transcriptase (Epicentre, Madison, WI, USA) was used in accordance with the recommended assay protocol provided by the manufacturer on the manual of the assay kit to convert the extracted RNA into cDNA, which was used in conjunction with a Hotstar Taq Polymerase real time PCR kit (Cat.no. AB-RPP-0005; Geneup, Shenzhen, China) on a StepOnePlus thermal cycler (ABI, Foster City, CA, USA) to carry out the Real-time PCR reactions. Finally, the relative expression of lncRNA-HOXA11-AS, miR-15a, TNF-α, and *NF-κB* in each sample was calculated based on the Ct value of amplification curves using the 2^−ΔΔCt^ approach.

### Cell culture and transfection

THP-1 cells, a human leukemia monocytic cell line, were obtained from ATCC (Manassas, VA, USA) and cultured in a RPMI 1640 medium (Corning, Corning, NY, USA) added with 10% of FBS (Gibco, Thermo Fisher Scientific, Waltham, MA) as well as 1% of PSG (penicillin, streptomycin, glutamine). The culture conditions were 37°C, saturated humidity and 5% carbon dioxide. In this study, in order to further explore the regulatory relationship among lncRNA-HOXA11-AS, miR-15a and STAT3, P-HOXA11-AS-A and P-HOXA11-AS-T were transfected into THP-1 cells, respectively. When the THP-1 cells reached 70% confluence, they were divided into 3 groups, i.e., 1. NC group (THP-1 cells transfected with a negative control); 2. P-HOXA11-AS-A group (THP-1 cells transfected with plasmids carrying P-HOXA11-AS-A); and 3. P-HOXA11-AS-T group (THP-1 cells transfected with plasmids carrying P-HOXA11-AS-T). The transfection was carried out using Lipofectamine 2000 (Invitrogen, Carlsbad, CA, USA) in accordance with the recommended transfection protocol provided on the manual of the transfection reagent. At 48 h post transfection, the cells were harvested to assay the expression of target genes of miR-15a and STAT3.

### Vector construction, mutagenesis and luciferase assay

Our online binding site screening process showed that miR-15a could potentially target lncRNA-HOXA11-AS (by searching https://circinteractome.nia.nih.gov/index.html) and STAT3 (by searching https://www.targetscan.org/vert_80/). To verify the regulatory relationship of lncRNA-HOXA11-AS, miR-15a and STAT3, the wild-type sequences of lncRNA-HOXA11-AS and STAT3 containing the miR-15a binding sites were cloned into pcDNA vectors (Promega, Madison, WI, USA) to generate wild type plasmids of lncRNA-HOXA11-AS and STAT3, respectively. In the next step, site-directed mutagenesis was carried out by using a Quick Change II mutagenesis assay kit (Stratagene, San Diego, CA, USA) according to the manufacture’s instruction to induce site-directed mutations in the miR-15a binding sites on lncRNA-HOXA11-AS and STAT3, and the mutant sequences of lncRNA-HOXA11-AS and STAT3 were also cloned into pcDNA vectors to generate mutant type plasmids of lncRNA-HOXA11-AS and STAT3, respectively. Then, using Lipofectamine 2000 (Invitrogen, Carlsbad, CA, USA) in accordance with the recommended transfection protocol provided on the manual of the transfection reagent, THP-1 cells were co-transfected with wild type or mutant lncRNA-HOXA11-AS and STAT3 plasmids in conjunction with miR-15a mimics, and the luciferase activity of transfected cells was evaluated by making use of a Dual luciferase reporter assay kit (Promega, Madison, WI, USA) at 48 post transfection.

### Western blot analysis

The total protein content in each sample was extracted by using a RIPA lysis buffer (Haigene, Harbin, China) containing 10 mM Tris/HCl, pH 7.4, 0.5% Triton X-100, 150 mM NaCl, and protease inhibitors according to the manufacturer’s instructions. In the next step, the extracted protein in each sample was separated on a 10% SDS-PAGE gel and blotted onto a nitrocellulose membrane, which was then blocked at room temperature for 2 h with 5% skim milk and probed with primary and secondary anti-STAT3 antibodies in sequence (Abcam, Cambridge, MA, USA) according to the recommended antibody incubation conditions provided by the manufacturer. After the membrane was washed with a TBS buffer (Sigma Aldrich, St. Louis, MO, USA) and developed by using an ECL reagent (Thermo Fisher Scientific, Waltham, MA, USA), and the density of the STAT3 protein bands on the membrane were assayed by using a Bio-Rad imaging system (Bio-Rad Laboratories, Hercules, CA, USA) to calculate the relative expression of STAT3 protein in each sample.

### ELISA

The abundance of STAT3, TNF-α, and NF-κB in collected peripheral blood samples was measured by using commercial ELISA assay kit (Thermo Fisher Scientific, Waltham, MA, USA) in accordance with the assay protocol provided on by the kits. The absorbance of samples at specific wavelengths was measured on a Multiskan GO microplate reader (Thermo Fisher Scientific, Waltham, MA, USA) in accordance with the recommended operating protocol provided by the instrument manufacturer.

### Statistical analysis

Statistical analyses were carried out by making use of SPSS 19.0 software (IBM, Chicago, IL, USA). One-way ANOVA and Student’s *t* tests were used to compare inter-group differences. All statistical tests were two-sided, while the *p*-values of < 0.05 indicated statistically significant.
